# Surgical gestures to evaluate apical dissection of robot-assisted radical prostatectomy

**DOI:** 10.1007/s11701-024-01902-0

**Published:** 2024-06-07

**Authors:** Maxwell X. Otiato, Runzhuo Ma, Timothy N. Chu, Elyssa Y. Wong, Christian Wagner, Andrew J. Hung

**Affiliations:** 1 Catherine and Joseph Aresty Department of Urology, Center for Robotic Simulation and Education, USC Institute of Urology, University of Southern California, Los Angeles, CA USA; 2 Department of Urology, Cedars-Sinai Medical Center, Los Angeles, CA USA; 3 Department of Urology and Urologic Oncology, St. Antonius-Hospital, Gronau, Germany

**Keywords:** Robotic surgery, Surgeon assessment, Dissection gestures, Apical dissection, Continence

## Abstract

Previously, our group established a surgical gesture classification system that deconstructs robotic tissue dissection into basic surgical maneuvers. Here, we evaluate gestures by correlating the metric with surgeon experience and technical skill assessment scores in the apical dissection (AD) of robotic-assisted radical prostatectomy (RARP). Additionally, we explore the association between AD performance and early continence recovery following RARP. 78 AD surgical videos from 2016 to 2018 across two international institutions were included. Surgeons were grouped by median robotic caseload (range 80–5,800 cases): less experienced group (< 475 cases) and more experienced (≥ 475 cases). Videos were decoded with gestures and assessed using Dissection Assessment for Robotic Technique (DART). Statistical findings revealed more experienced surgeons (*n* = 10) used greater proportions of *cold cut* (*p* = 0.008) and smaller proportions of peel/push, spread, and two-hand spread (*p* < 0.05) than less experienced surgeons (*n* = 10). Correlations between gestures and technical skills assessments ranged from − 0.397 to 0.316 (*p* < 0.05). Surgeons utilizing more *retraction* gestures had lower total DART scores (*p* < 0.01), suggesting less dissection proficiency. Those who used more gestures and spent more time per gesture had lower efficiency scores (*p* < 0.01). More coagulation and hook gestures were found in cases of patients with continence recovery compared to those with ongoing incontinence (*p* < 0.04). Gestures performed during AD vary based on surgeon experience level and patient continence recovery duration. Significant correlations were demonstrated between gestures and dissection technical skills. Gestures can serve as a novel method to objectively evaluate dissection performance and anticipate outcomes.

## Introduction

Growing evidence shows that surgical performance and skill impact postoperative patient outcomes [1]. However, objectively quantifying this performance remains a challenge within surgical education. Historically, the surgical learning environment has relied on sheer repetition and qualitative feedback from human experts. This educational paradigm continues to evolve and shift towards a more competency-based model [2]. As it stands, technical skill assessments, such as Dissection Assessment for Robotic Technique (DART) [3], are the most common method for grading fundamental surgical skills. However, such tools are based on reviewer observation and some subjective judgment, which can be prone to bias [4].

**Fig. 1:**
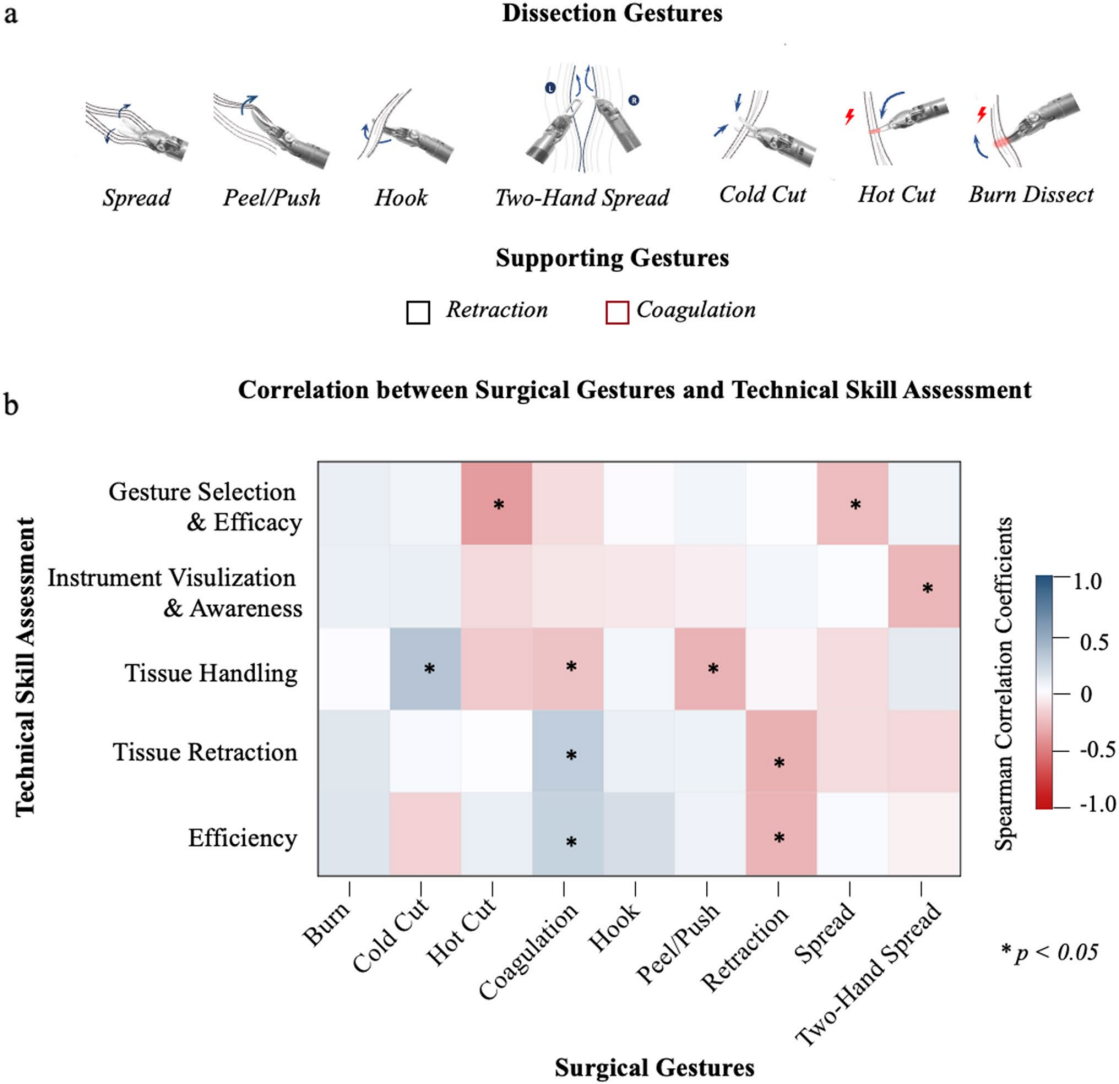
Correlation between surgical Gestures and Technical Skills in Apical Dissection

Surgical education is in need of a reliable and objective method that assesses surgical skills to enhance training curricula and outcomes. Surgical gestures, fundamental interactions of surgical instruments with human tissue, present a novel approach to deconstructing surgery [5]. Previously, our group introduced a gesture classification system involving nine dissection gestures (e.g., hook) and four supporting gestures (e.g., retraction) (Fig. 1a) [6]. Gesture selection has been found to measure performance and influence outcomes in a specific surgical step of robot-assisted radical prostatectomy (RARP) [7].

While the visualization and preservation of anatomy in the apical dissection (AD) step have been associated with postoperative urinary continence [8], the relationship between apical dissection performance—as measured by gestures—and continence recovery remains to be explored. In this study, we evaluate gesture usage as a performance measure by linking them with surgeon experience and skill assessment scores in AD of RARP. The aims were to (1) assess variations in gesture usage among surgeons of different experience levels, (2) correlate gesture usage with existing technical skills assessment tools, and (3) investigate whether AD performance (gesture usage and technical skills) is linked to the early recovery of urinary continence after RARP.

## Materials and methods

### Study cohort and design

This prospective study was approved by the University of Southern California institutional review board. RARPs with AD video footage from July 2016 to November 2018 from two international institutions were included in the study. The cases were performed by surgical fellows and faculty surgeons. Surgeons were separated into two experience levels by the median robotic caseload. The less experienced and more experienced groups were defined by < 475 and ≥ 475 robotic cases, respectively. The functional outcome of interest is urinary continence recovery at three months following RARP. Urinary continence was defined as the absence of pads or the use of one safety per day, which remains dry for the majority of the time [9].

### Video annotation and rating

Dissection performance from a recorded AD step of the RARP cases was manually reviewed. Videos were blindly annotated using a previously published surgical gesture classification system and assessed using the DART tool.

For surgical gestures, two annotators (MO, RM) received standardized training and independently labeled gesture sequences of 3 training videos for a total of 365 gestures. Each annotation included the name of the gesture, the duration, and the arm in which the gesture was performed (i.e., left or right). The gesture classification agreement rate between the annotators was calculated as the proportion of gesture labels agreed upon in the total number of gestures. An inter-rater agreement rate of 91.2% was achieved. 78 AD videos were split and annotated between the annotators. For technical skills assessment scoring, raters (MO, RM) received standardized training and independently rated 78 AD videos using DART. Inter-rater reliability of DART domains for a batch of four cases showed 90% observed agreement. A session was held after scoring to discuss the consensus and discrepant scoring of the reviewed videos. DART scores were finalized after discussion.

### Statistical analysis

Statistical analysis was conducted using IBM SPSS v24, with a statistical significance set at *p* < 0.05. A Kruskal–Wallis test was used to compare surgical gesture proportion with DART scores from surgeons of different experience levels. Spearman’s correlation coefficients were used to assess the associations between surgical gesture usage and DART. Mann–Whitney *U* tests were performed to compare the distribution of gesture usage among patients grouped by continence recovery status at the 3-month postoperative period.

## Results

78 cases from surgeons from 20 surgeons across two international surgical centers were included in the analysis. The median prior robotic surgical caseload of the 20 surgeons was 475 (range 80–5839) cases. Ten surgeons were defined as more experienced, while 10 surgeons were less experienced by the median caseload split.

Active dissection gestures made up 68.8% of total gestures, while supporting gestures consisted of the remaining 31.2% (Table 1). *Cold cut* was the most commonly performed gesture (42.5%), followed by peel/push (21.6%). More experienced surgeons used greater proportions of cold cut compared to less experienced surgeons (average 75.14% vs. 72.55%, *p* < 0.05). More experienced surgeons also had smaller proportions of peel/push, spread, and two-hand spread than less experienced surgeons (average 43.9% vs. 58.7% for peel/push, 1.5% vs. 12.5% for spread, 0% vs. 3.76% for two-hand spread, *p* < 0.05).

**Table 1:** Comparing gesture proportion by distribution across patient and surgeon groups in AD

Gestures	Proportion of total gestures (%)	Correlation between gestures and continence groups* (*p* Value)	Correlation between gestures and surgeon experience levels* (*p* Value)
Peel/Push	21.6	0.276	**0.02**
Cold cut	42.5	0.108	**0.008**
Spread	0.5	0.267	**0.001**
Hook	1.5	**0.036**	0.144
Hot Cut	1.2	0.282	0.353
Two-hand spread	0.2	0.207	**0.044**
Burn	0.1	0.207	0.75
Retraction	8.9	0.207	0.6
Coagulation	1.2	**0.003**	0.091

Bold values are statistically significant (*p* Value ≤ 0.05)

*p-values are associated with test statistics for Mann–Whitney *U*-tests assessing patient 3-month continence recovery status and surgeon experience levels (split by median robotic caseload of surgeons, 475 cases)

Five domains of the technical skills assessment tool DART were assessed. More experienced surgeons were found to have significantly higher Gesture Selection, Tissue Retraction, and total DART scores than less experienced surgeons (*p* < 0.05, Table 2).

**Table 2:** Association between DART Scores during AD and Surgeon Experience Levels (split by median robotic caseload of surgeons, 475 cases)

DART domain*	Less experienced surgeons (*n* = 10) 18 cases in total mean ± SD^#^	More experienced surgeons (*n* = 10) 60 cases in total mean ± SD^#^	*p* Value^#^
Gesture selection and efficacy	**2.7 ± 0.5**	**3.0 ± 0.2**	**0.007**
Instrument visualization and awareness	2.9 ± 0.2	2.9 ± 0.4	0.62
Tissue handling	2.5 ± 0.7	2.7 ± 0.6	0.26
Tissue retraction	**2.6 ± 0.7**	**2.8 ± 0.4**	**0.042**
Efficiency	2.1 ± 0.8	2.4 ± 0.7	0.10
Total DART scores	**12.8 ± 1.7**	**13.8 ± 1.1**	**0.010**

Bold values are statistically significant (*p* Value ≤ 0.05)

*Only five domains of DART were chosen to be used for apical dissection. Respect for tissue plane domain does not apply to this step

^#^Mean ± SD and *p*-value were calculated using a mixed-effect model to adjust for data clustering within surgeons

*p-values are associated with test statistics for Mann–Whitney *U*-tests assessing patient 3-month continence recovery status and surgeon experience levels (split by median robotic caseload of surgeons, 475 cases)

Associating gestures with the domains of DART showed multiple positive and negative relationships. Correlations between the surgical gestures and technical skills assessments ranged from -0.397 to 0.316 using Spearman’s correlation coefficient (*p* < 0.05, Fig. 1b). The total count of gestures and time spent per gesture was significantly associated with the Efficiency domain (*p* < 0.01). Total DART scores were significantly associated with the proportion of retraction gestures (*p* < 0.01).

Three-month postoperative continence recovery rate was 55% (43/78). Patients with continence recovery had significantly higher coagulation and hook gesture usage than patients with ongoing postoperative incontinence (average usage 5.85 vs. 1.3 for coagulation, 3.0 vs. 2.3 for hook, *p* < 0.04). No statistically significant associations were found between DART domains and time to continence recovery.

## Discussion

This study demonstrated that: (1) surgical gesture selections are associated with surgeon experience; (2) gestures are linked with dissection technical skill assessment scores; (3) different proportions of gesture usage are associated with faster outcome recovery. This suggests that gestures can be considered a valid and objective metric for evaluating surgical proficiency.

Notably, surgeons of different experience levels use different surgical gestures during AD, as evidenced by the variable usage of dissection gestures. These findings are consistent with the results of previous studies [7]. The use of distinct gesture selections and patterns can provide a measure of a surgeon’s skill set. Understanding how expert surgeons select certain gestures can guide the development of training protocols for novice surgeons and trainees.

This investigation revealed that select gesture usage is associated with 3-month continence recovery following RARP. The results indicate that greater usage of hook and coagulation was employed during surgeries for patients with shorter continence recovery. Considering the anatomic variation observed in the prostatic apex [8], we posit that hook usage may enhance precision, whereas coagulation usage may improve visibility during AD, ultimately contributing to more favorable surgical outcomes. These findings imply that the selection of gestures and surgical performance of AD can help in identifying postoperative risk or optimizing postoperative outcome recovery.

The study establishes a link between dissection gestures and the technical skills domains assessed by DART. This finding further supports the utility of gestures as a novel approach to evaluating surgical performance. Integrating both gesture and technical skills assessments offers a holistic understanding of a surgeon's skill set and proficiency in the operating room. Furthermore, these results underline the advantages of gestures over technical skill assessment tools. Conventional skill assessment tools, such as DART, offer a broad evaluation of a surgeon's capabilities, reflecting skills at a general procedural level. In contrast, gestures deconstruct operations to discrete moment-to-moment instrument-human tissue interactions, reflecting a more granular level of insight [5]. While both metrics are interconnected, gestures can provide immediate explainable, and actionable teaching points that can be imparted to trainees.

Together these findings suggest gestures present specific, actionable opportunities for training and instruction. In addition to previous studies, these findings affirm that gestures are reliable, specific, and objective. Future work should move toward establishing gestures as a bridge connecting machine learning-based assessment tools to real-time, interpretable teaching points of clinical value for surgical trainees.

## Limitations

These findings should be considered in the context of certain limitations. The study faced constraints in recruiting multiple raters for technical skill assessment scoring, potentially affecting the comprehensiveness and accuracy of scores. While the study was bi-institutional to enhance generalizability, the sample size of included cases was relatively small and may have introduced bias. Additionally, the binary classification of surgeons based on two experience levels does not fully reflect the intricacies of a surgical learning curve and the case-by-case varying performances. It is important to note that future research should assess the broader application of gestures across various procedures and specialties, considering different levels of training. Despite these limitations, these findings offer insight into the ongoing evolution of surgical education toward curricula centered on skill assessment.

## Conclusion

The findings of this study highlight that surgeons with varying experience levels employ different surgical gestures during AD. This considerable variation—as measured by gestures—may offer a detailed and objective means for evaluating both AD performance and urinary continence recovery. These findings collectively suggest that deconstructing procedures to gestures can serve as a reliable basis for teaching and evaluating dissection skills. Gestures can objectively quantify surgical skills to improve the performance of trainees.

## Data Availability

No datasets were generated or analysed during the current study.
